# Supplementary material for: Bayesian estimation of Lassa virus epidemiological parameters: Implications for spillover prevention using wildlife vaccination
Source: PLoS Negl Trop Dis. 2020 Sep 21;14(9):e0007920. doi: 10.1371/journal.pntd.0007920 (PMC7529244; doi:10.1371/journal.pntd.0007920)
Supplement: S1 Table — (PDF) [file pntd.0007920.s001.pdf]

Supplemental Table 1. Summary statistics for *M. natalensis* life history traits calculated for 18 breeding groups in a captive colony from Mali

	Age at First Reproduction in weeks (Dam)	Litter Size	Age at Weaning (days)	Inter-Litter Interval (days)
Number of values	17	84	84	58
Minimum	12	3	14	20
25% Percentile	17	9	17	23
Median	20	11	20	24
75% Percentile	21.5	13	22	26
Maximum	23	15	30	31
Mean	18.82	10.74	19.61	24.26
Std. Deviation	3.302	2.685	3.293	2.181
Std. Error of Mean	0.8009	0.2929	0.3593	0.2864
Lower 95% CI of mean	17.13	10.16	18.89	23.69
Upper 95% CI of mean	20.52	11.32	20.32	24.83
