# Supplementary material for: Bayesian estimation of Lassa virus epidemiological parameters: Implications for spillover prevention using wildlife vaccination
Source: PLoS Negl Trop Dis. 2020 Sep 21;14(9):e0007920. doi: 10.1371/journal.pntd.0007920 (PMC7529244; doi:10.1371/journal.pntd.0007920)
Supplement: S2 Table — Rows do not always sum to totals due to gaps in viral diagnostics that prevented individuals from being assigned to a category. (PDF) [file pntd.0007920.s002.pdf]

S2_Table. Rodent capture data stratified by age class and infection status. Rows do not always sum to totals due to gaps in viral diagnostics that prevented individuals from being assigned to a category.

Table S2A. Rodent capture data for Bantou								
	Juveniles			Adults			Total Juveniles	Total Adults
Date	PCR – IgG -	PCR +	PCR – IgG +	PCR – IgG -	PCR +	PCR – IgG +		
Oct-03	23	12	5	9	4	11	43	24
Jan-04	13	2	0	12	0	2	32	29
May-04	13	5	2	15	5	4	22	36
Oct-04	19	7	6	9	3	8	33	20
Jan-05	--	--	--	--	--	--	36	28

Table S2B. Rodent capture data for Tanganya								
	Juveniles			Adults			Total Juveniles	Total Adults
Date	PCR – IgG -	PCR +	PCR – IgG +	PCR – IgG -	PCR +	PCR – IgG +		
Oct-03	23	6	14	10	5	15	43	30
Jan-04	8	3	3	4	1	6	22	15
May-04	5	1	0	1	7	4	14	20
Oct-04	21	1	2	4	2	7	25	13
Jan-05	--	--	--	--	--	--	30	19
