# Supplementary material for: Bayesian estimation of Lassa virus epidemiological parameters: Implications for spillover prevention using wildlife vaccination
Source: PLoS Negl Trop Dis. 2020 Sep 21;14(9):e0007920. doi: 10.1371/journal.pntd.0007920 (PMC7529244; doi:10.1371/journal.pntd.0007920)
Supplement: S1 Text — (DOCX) [file pntd.0007920.s003.docx]

S3_Text

*Part 1: Evaluating performance of ABC approach using simulated data*

Before applying our ABC method to real data, we evaluated its performance by testing against simulated data sets. Simulated data sets for the two Guinean villages were generated by: 1) drawing parameters at random from the prior distributions shown in Table 2 of the main text, 2) simulating population and epidemiological dynamics in these populations using the Gillespie algorithm and equations (1-2) with local precipitation data for each village from the CHIRPS 2.0 database, and 3) conducting simulated rodent trapping experiments. Simulated rodent trapping was conducted on the same dates as in the real dataset and the number of trap nights in each session was drawn at random from a uniform distribution on [800, 1200] to bracket values in the real data. The trapping rate parameter $\tau$ was set to $3.5\times{10}^{-5}$ for simulating data sets, a rate that yields biologically plausible values for true population density of *M. natalensis* within the Guinean villages that range between $\approx600-2,000$ post-weaning and trappable animals per village. In total, we generated 30 simulated data sets, each of which was then subjected to analysis using our ABC methodology.

To maximize the number of synthetic data sets we could analyze, we applied our ABC algorithm to each synthetic data set until at least 1000 points were accumulated in the posterior distribution. The small size of the posterior distribution used in our simulation testing should yield conservative estimates for how well our methodology is likely to work when applied to real data and run until a much larger number of points are accumulated in the posterior distribution. Once the ABC algorithm had been applied to each synthetic data set, we calculated the mode and 95% credible interval of the marginal posterior distribution for each model parameter, and for the composite parameter $R_{0}$.

Comparison of parameter values in the simulated data and the values inferred by our ABC methodology showed that the data contains strong signal for only five model parameters. Specifically, the correlation between estimated and simulated parameter values exceeds 0.5 for only the following parameters: 1) the rate of horizontal transmission ($\beta$), 2) the rate of recovery from viral infection $\left( \gamma\right)$, 3) the strength of density dependent mortality in each population ($k_{X}$), and 4) the sensitivity of birth rates to past precipitation ($\rho$) (Figure S1). In addition to these individual parameters, our analysis shows that the composite parameter $R_{0}$ is estimated quite accurately by our approach (Figure 2 of main text). Remaining parameters showed no meaningful association between true and estimated values suggesting they may not be identifiable using the available data and summary statistics.

*Part 2: Calculating time-averaged* $R_{0}$

We calculated the time-averaged value of $R_{0}$ using the next generation method and the density of susceptible individuals at each time point in the simulation [1]. To calculate the $R_{0}$ of system (1) at a single geographic location using the next-generation method, we rewrite equations (1d—f) as $\dot{I}_{i}=F_{i}-W_{i}$ for $i\in\{P,J,A\}$ where $F_{i}$ gives the rate of new infections and $W_{i}$ gives the rate of change of $I_{i}$ by means other than new infections for age class $i$. This yields the following equations for $F_{i}$ and $W_{i}$:

$F_{P}=bVI_{A}\left( 1-\varphi N \right)$

$F_{J}=\beta\left( I_{A}+I_{J} \right)S_{J}$

$F_{A}=\beta\left( I_{A}+I_{J} \right)S_{A}$

$W_{P}=\left( \gamma+\alpha_{PJ}+d_{P}+kN \right)I_{P}$

$W_{J}=-\alpha_{PJ}I_{P}+\left( \gamma+\alpha_{JA}+d_{J}+kN \right)I_{J}$

$W_{A}=-\alpha_{JA}I_{J}+\left( \gamma+d_{A}+kN \right)I_{A}$

where $N=S_{P}+S_{J}+S_{A}+I_{P}+I_{J}+I_{A}+R_{P}+R_{J}+R_{A}$.

Let $N_{0}=\left( N_{P},N_{J},N_{A} \right)^{T}$ be the disease free equilibrium, in which all individuals are susceptible, and define the matrices $F=\frac{\partial F_{i}}{\partial I_{j}}(N_{0})$ and $W=\frac{\partial W_{i}}{\partial I_{j}}(N_{0})$ for $i,j\in\{P,J,A\}$. The next-generation matrix is given by $FW^{-1}$. The spectral radius of $FW^{-1}$ is the basic reproductive number:

$$R_{0}=(\alpha_{JA}\left( \beta\left( \gamma+kN+d_{P} \right)\left( N_{A}+N_{J} \right)+\alpha_{PJ}\left( bV+\left( \beta-bV\phi\right)\left( N_{A}+N_{J} \right)-bV\phi N_{P} \right) \right)+$$

$$\beta\left( \gamma+kN+d_{P}+\alpha_{PJ} \right)\left( kN_{A}^{2}+N_{A}\left( \gamma+d_{J}+2kN_{J}+kN_{P} \right)+N_{J}\left( \gamma+d_{A}+k\left( N_{J}+N_{P} \right) \right) \right)+$$

$$((-4bV\beta\left( N\phi-1 \right)\left( \gamma+kN+d_{A} \right)\left( \gamma+kN+d_{J}+\alpha_{JA} \right)\alpha_{PJ}\left( \gamma+kN+d_{P}+\alpha_{PJ} \right)N_{A}+$$

$$(\alpha_{JA}\left( \beta\left( \gamma+kN+d_{P} \right)\left( N_{A}+N_{J} \right)+\alpha_{PJ}\left( bV+\left( \beta-bV\phi\right)\left( N_{A}+N_{J} \right)-bV\phi N_{P} \right) \right)+$$

$$\beta(\gamma+kN+d_{P}+\alpha_{PJ})(kN_{A}^{2}+N_{A}\left( \gamma+d_{J}+2kN_{J}+kN_{P} \right)+N_{J}\left( \gamma+d_{A}+k\left( N_{J}+N_{P} \right) \right)))^{2})^{\frac{1}{2}})/$$

$$(2\left( \gamma+kN+d_{A} \right)\left( \gamma+kN+d_{J}+\alpha_{JA} \right)\left( \gamma+kN+d_{P}+\alpha_{PJ} \right))$$

where $N=N_{P}+N_{J}+N_{A}$.

An $R_{0}$ value was calculated at each time point for each simulation in the posterior by plugging in the parameter values and the time-varying number of individuals in each age class $N_{i}$ for $i\in\{P,J,A\}$ assuming that all individuals are susceptible (the definition of $R_{0}$). The time-averaged $R_{0}$ was then calculated by averaging these values for each simulation in the posterior.

*Part 3: Results for density dependent birth*

Previous work exploring the population dynamics of *M. natalensis* has demonstrated the importance of density dependent mortality but not ruled out a role for density dependent birth. Because the way in which density dependence acts may alter the scope for effective vaccination, we consider the alternative scenario of density dependent birth here. Our methodology is identical to the case of density dependent mortality described in the main text, with the only difference being that the strength of density dependent mortality $\left( k_{x} \right)$ is set to zero and the strength of density dependent birth $\left( \varphi_{x} \right)$ drawn from a uniform prior distribution on [$0.0004, 0.0018$]. This range was chosen to generate biologically reasonable rodent population sizes.

We applied our ABC method under the assumption of density dependent birth until 12,609 points were accumulated in the posterior distribution. Posterior distributions were well-resolved, with estimates for R0 similar to those derived under the assumption of density dependent mortality (Supplemental Figure 2). We then used this posterior distribution to simulate vaccination campaigns otherwise identical to those described in the main text. The results demonstrate that density dependent birth is more favorable to the success of vaccination campaigns. The likely reason for this difference is that the reduced birth rates slow the dilution of immunity provided by vaccination.

References

1. Diekmann O, Heesterbeek JAP, Metz JAJ. On the definition and the computation of the basic reproduction ratio R_0_ in models for infectious-diseases in heterogeneous populations. Journal of Mathematical Biology. 1990;28(4):365-82. PubMed PMID: WOS:A1990DG35000001.


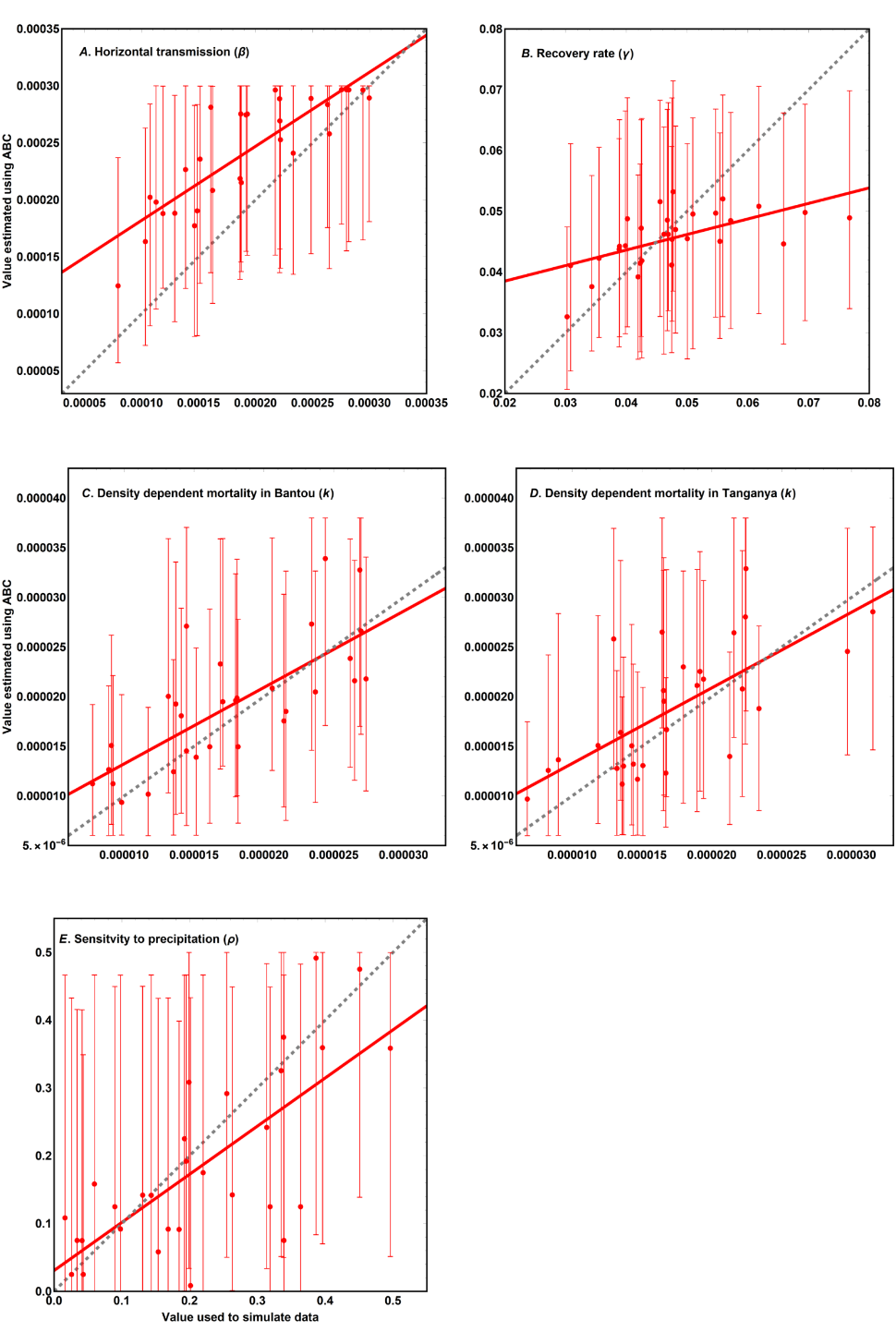


Figure S1. Comparison of parameters used to generate simulated data sets and the values for these parameters estimated by our ABC method. Results are shown for only those parameters exhibiting strong positive correlations (> 0.5) between true and estimated values. Dots indicate the mode of the marginal posterior distributions and intervals represent 95% credible intervals for the marginal posterior distributions. The solid red line indicates the best linear fit of estimated and true values and the dashed black line the expected 1:1 relationship for a perfect fit.


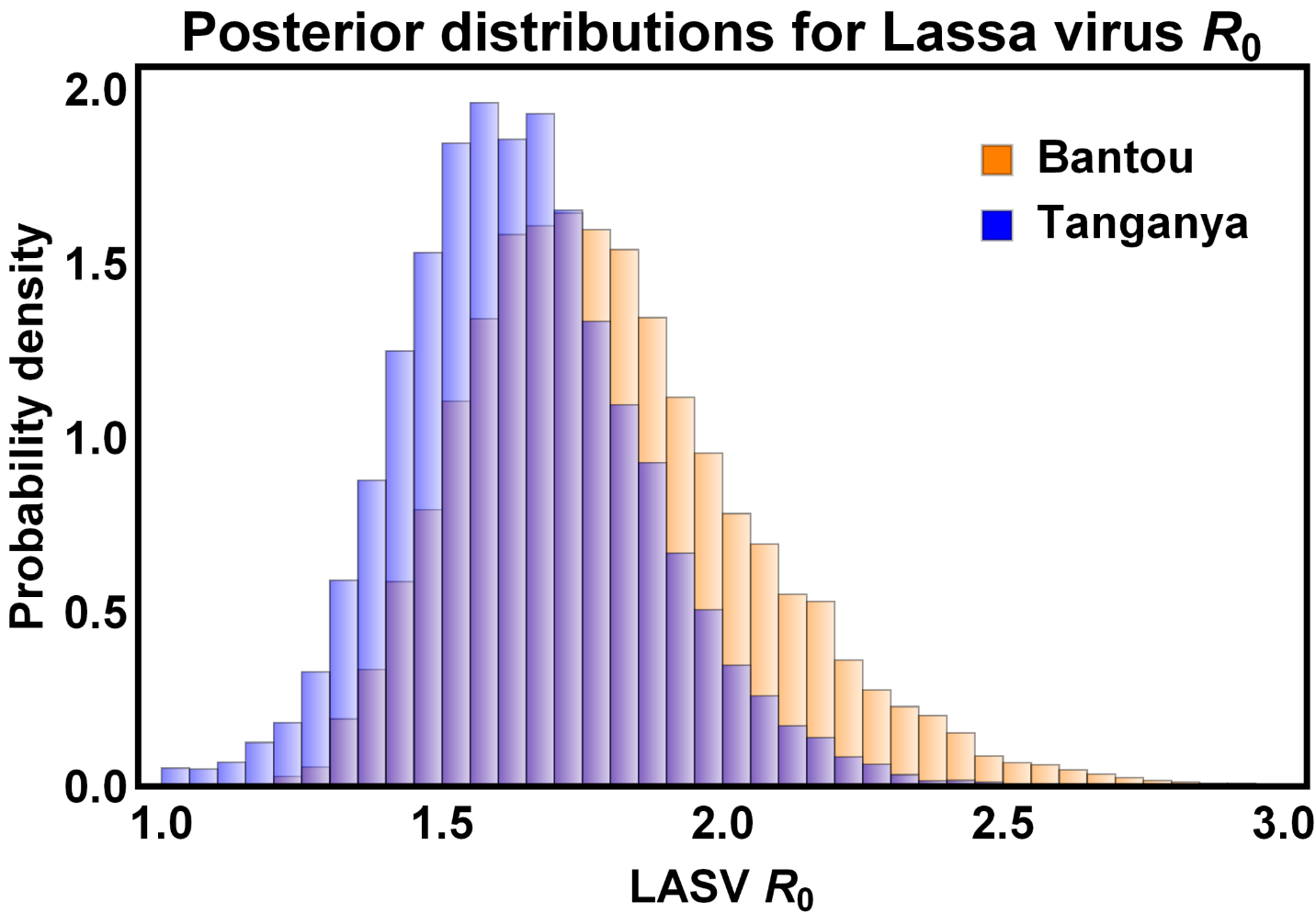


Figure S2. Posterior distributions for $R_{0}$ in the villages of Bantou and Tanganya under the assumption that density dependence acts on birth rates rather than mortality rates. The modal values and credible intervals for $R_{0}$ varied somewhat across villages, with modal values of 1.75 and 1.57 and credible intervals of {1.29, 2.21} and {1.09, 1.91} in Bantou and Tanganya, respectively.
