# Supplementary figures and images for: Bayesian estimation of Lassa virus epidemiological parameters: Implications for spillover prevention using wildlife vaccination
Source: PLoS Negl Trop Dis. 2020 Sep 21;14(9):e0007920. doi: 10.1371/journal.pntd.0007920 (PMC7529244; doi:10.1371/journal.pntd.0007920)

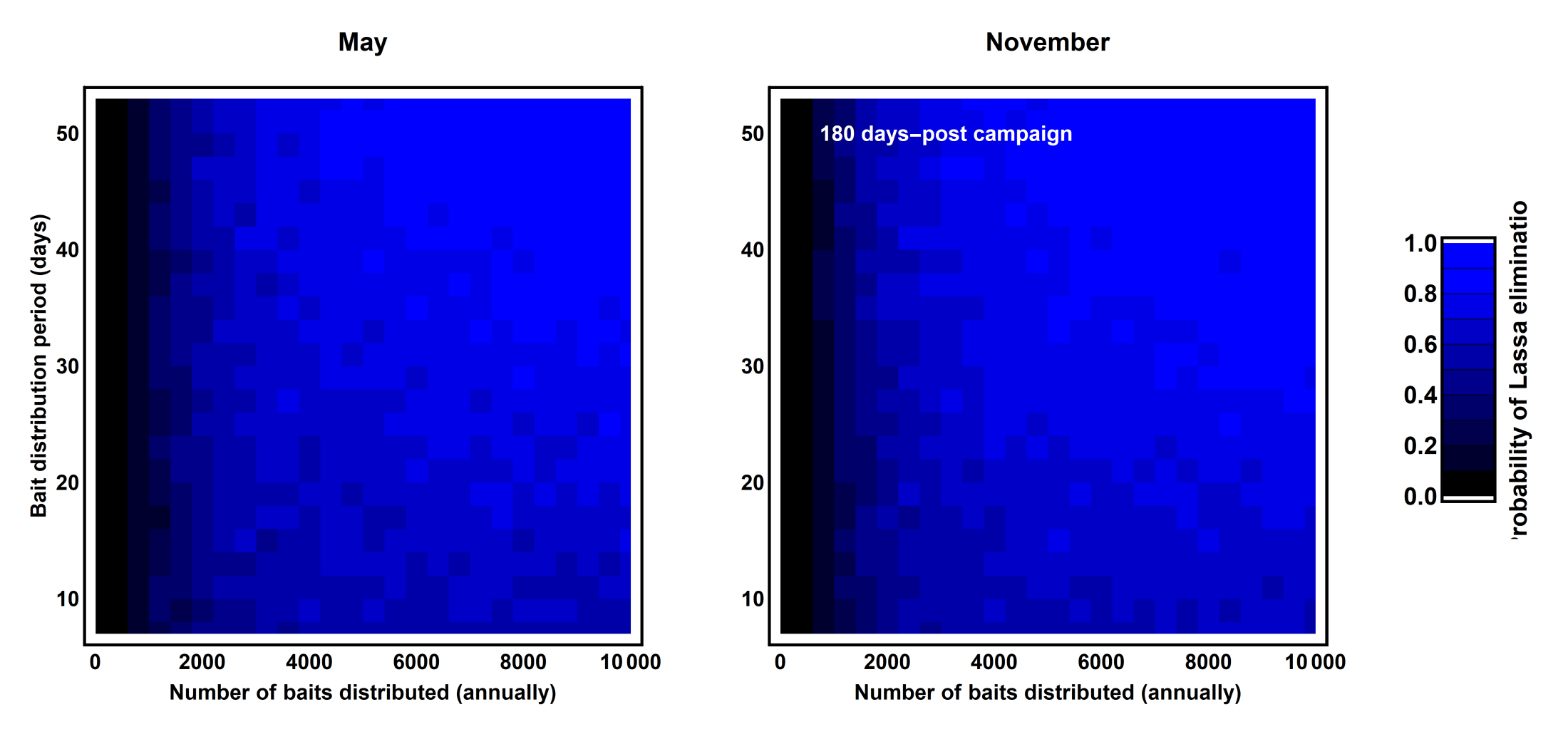

Supplement: S1 Fig — The proportion of simulated vaccination campaigns resulting in the simultaneous elimination of Lassa virus from the villages of Bantou and Tanganya as a function of the number of vaccine laced baits distributed per year (x axis) and the duration of bait distribution (y axis). The left-hand column shows results for campaigns where vaccination occurs in May when birth rates are minimized (bait distribution begins May 1 of each year). and the right hand column campaigns where vaccination occurs in November when birth rates are maximized (bait distribution begins November 1 of each year). The model used to generate this figure assumed density dependence acts on birth rather than death as in the main text. (TIF) [file pntd.0007920.s004.tif]
